# 12,12′-[2,2′-Oxybis(ethane-2,1-di­yl)bis­(­oxy)]bis­[(*R*_p_)-4-bromo­[2.2]paracyclo­phane]

**DOI:** 10.1107/S1600536811010051

**Published:** 2011-03-23

**Authors:** Bing Hong, Yudao Ma, Wenzeng Duan, Fuyan He, Lei Zhao

**Affiliations:** a School of Chemistry and Chemical Engineering, Shandong University, Jinan 250100, People’s Republic of China

## Abstract

The title compound, C_36_H_36_Br_2_O_3_, was synthesized from (*R*_p_)-4-bromo-12-hy­droxy[2.2]paracyclo­phane and oxydiethane-2,1-diyl bis­(4-methyl­benzene­sulfonate). The crystal packing exhibits a short O⋯Br inter­action [Br⋯O = 3.185 (3) Å] and a weak inter­molecular C—H⋯O contact.

## Related literature

The title compound is an important inter­mediate in the application of paracyclo­phanes, especially used as ligands in asymmetric catalysis. For the structure of [2.2]paracyclo­phane, see: Singer & Cram (1963 ▶); Gibson & Knight (2003 ▶); Rivera *et al.* (2011 ▶). For bis­(diphenyl­phosphino)-[2.2]paracyclo­phane, see: Pye *et al.* (1997 ▶). For the application of salen ligands based on [2.2]paracyclo­phane as asymmetic ligands, see: Dahmen & Bräse (2002 ▶); Bräse & Höfener (2005 ▶); Lauterwasser *et al.* (2006 ▶). For the synthesis of (*R*_p_)-4-bromo-12-hy­droxy[2.2]paracyclo­phane, see: Jiang & Zhao (2004 ▶).
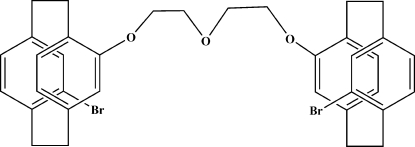


## Experimental

### 

#### Crystal data

C_36_H_36_Br_2_O_3_
*M* *_r_* = 676.47
Orthorhombic, 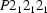

*a* = 8.850 (4) Å
*b* = 12.019 (5) Å
*c* = 28.242 (12) Å
*V* = 3004 (2) Å^3^
*Z* = 4
Mo *K*α radiation
μ = 2.73 mm^−1^
*T* = 273 K
0.13 × 0.12 × 0.10 mm

#### Data collection

Bruker APEXII CCD diffractometer
Absorption correction: multi-scan (*SADABS*; Bruker, 2007 ▶) *T*_min_ = 0.718, *T*_max_ = 0.772
12887 measured reflections
4331 independent reflections
3692 reflections with *I* > 2σ(*I*)
*R*_int_ = 0.030
θ_max_ = 23.3°

#### Refinement

*R*[*F* ^2^ > 2σ(*F* ^2^)] = 0.030
*wR*(*F* ^2^) = 0.068
*S* = 1.02
4331 reflections
370 parameters
H-atom parameters constrained
Δρ_max_ = 0.39 e Å^−3^
Δρ_min_ = −0.35 e Å^−3^
Absolute structure: Flack (1983 ▶), 1839 Friedel pairs
Flack parameter: 0.008 (8)

### 

Data collection: *APEX2* (Bruker, 2007 ▶); cell refinement: *SAINT* (Bruker, 2007 ▶); data reduction: *SAINT*; program(s) used to solve structure: *SHELXS97* (Sheldrick, 2008 ▶); program(s) used to refine structure: *SHELXL97* (Sheldrick, 2008 ▶); molecular graphics: *SHELXTL* (Sheldrick, 2008 ▶); software used to prepare material for publication: *SHELXTL*.

## Supplementary Material

Crystal structure: contains datablocks I, global. DOI: 10.1107/S1600536811010051/fy2003sup1.cif

Structure factors: contains datablocks I. DOI: 10.1107/S1600536811010051/fy2003Isup2.hkl

Additional supplementary materials:  crystallographic information; 3D view; checkCIF report

## Figures and Tables

**Table 1:** Hydrogen-bond geometry (Å, °)

*D*—H⋯*A*	*D*—H	H⋯*A*	*D*⋯*A*	*D*—H⋯*A*
C17—H17*A*⋯O2^i^	0.97	2.71	3.412 (5)	130

Symmetry code: (i) 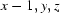.

**Fig. 1.**
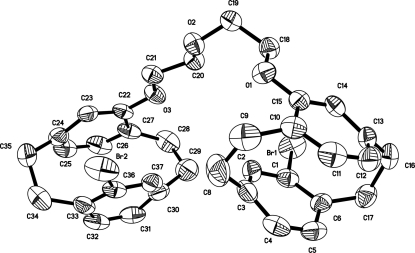
The molecular structure of (I) showing the atom numbering scheme and 50% probabilty displacement ellipsoids. H atoms are omitted for clarity.

**Fig. 2.**
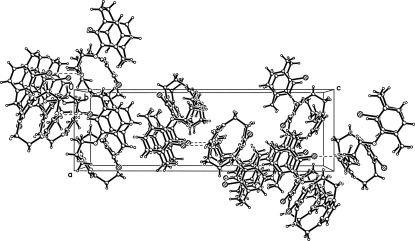
The superomolecular structure of (I), showing the intermolecular O···Br interaction and the weak C-H···O interaction.

## supplementary crystallographic information

### Comment

The chemistry of [2.2]paracyclophane gathered great attention since the middle of last century (Singer & Cram, 1963). When the position on the aryl group of paracyclophane was substitued, [2.2]paracyclophane presented planar chirality due to its conformationally rigid structure. After 4,12-bis(diphenylphosphino)-[2.2]paracyclophane was synthesized and applied in aymmetric hydrogenation (Pye *et al.*, 1997), the application of salen ligands based on [2.2]paracyclophane in asymmetic addition reations on aldehydes was exploited (Dahmen & Bräse, 2002; Bräse & Höfener, 2005; Lauterwasser *et al.*, 2006).

In the title compound (Fig. 1), the C—Br bond lengths are 1.903 (4) Å and 1.905 (3) Å, respectively, which are in agreement with the C—Br bond length of 1.9080 (16) Å reported by Rivera *et al.* (2011) for a 4-bromophenol derivative. The C(15)—O(1) bond [1.385 (4) Å] and the C(22)—O(3) bond [1.374 (4) Å] are longer than the similar C(ph)—O bond [1.353 (2) Å] of Rivera *et al.* (2011), which is due to the weaker p—π conjugation in our [2.2]paracyclophane backbone. The intermolecular C—H···O and O···Br contacts link the molecules into a polymeric tape structure (Fig. 2).

### Experimental

(*R*_p_)-4-bromo-12-hydroxy[2.2]paracyclophane (0.152 g, 0.50 mmol), which was prepared according to the published procedure (Jiang *et al.*, 2004), was dissolved in 5.0 ml DMF in a flask. Then oxydiethane-2,1-diyl bis(4-methylbenzenesulfonate) (0.108 g, 0.26 mmol) and K_2_CO_3_ (0.208 g, 1.50 mmol) were added. The flask was incubated at 353 K in oil bath for 8 h. After reaction, the reaction solution was filtered, then 20 ml water was added and the product was extracted with 10 ml CH_2_Cl_2_ (three times) and the organic phase was washed with 5 ml water (also three times). The CH_2_Cl_2_ was vacuum distilled and the crude product was subjected to column chromatography on silica gel. The yield of pure product was 0.106 g (68%) as a white solid. The colourless crystals suitable for an X-ray diffraction experiment were obtained by slow diffusion of *n*-hexane into a solution of the product in CH_2_Cl_2_.

### Refinement

All the H atoms were located in difference maps; H atoms bonded to C atoms were then treated as riding atoms in geometrically idealized positions, with C—H distances of 0.93 (aromatic) and 0.97 (aliphatic) Å and with U_iso_(H) = 1.2 U_eq_(C).

### Crystal data

**Table:**

C_36_H_36_Br_2_O_3_	*F*(000) = 1384
*M**_r_* = 676.47	*D*_x_ = 1.496 Mg m^−^^3^
Orthorhombic, *P*2_1_2_1_2_1_	Mo *K*α radiation, λ = 0.71073 Å
Hall symbol: P 2ac 2ab	Cell parameters from 4449 reflections
*a* = 8.850 (4) Å	θ = 1.8–23.3°
*b* = 12.019 (5) Å	µ = 2.73 mm^−^^1^
*c* = 28.242 (12) Å	*T* = 273 K
*V* = 3004 (2) Å^3^	Block, colourless
*Z* = 4	0.13 × 0.12 × 0.10 mm

### Data collection

**Table:**

Bruker APEXII CCD diffractometer	4331 independent reflections
Radiation source: fine-focus sealed tube	3692 reflections with *I* > 2σ(*I*)
graphite	*R*_int_ = 0.030
φ and ω scans	θ_max_ = 23.3°, θ_min_ = 1.8°
Absorption correction: multi-scan (*SADABS*; Bruker, 2007)	*h* = −9→7
*T*_min_ = 0.718, *T*_max_ = 0.772	*k* = −13→13
12887 measured reflections	*l* = −29→31

### Refinement

**Table:**

Refinement on *F*^2^	Secondary atom site location: difference Fourier map
Least-squares matrix: full	Hydrogen site location: inferred from neighbouring sites
*R*[*F*^2^ > 2σ(*F*^2^)] = 0.030	H-atom parameters constrained
*wR*(*F*^2^) = 0.068	*w* = 1/[σ^2^(*F*_o_^2^) + (0.0252*P*)^2^] where *P* = (*F*_o_^2^ + 2*F*_c_^2^)/3
*S* = 1.02	(Δ/σ)_max_ < 0.001
4331 reflections	Δρ_max_ = 0.39 e Å^−^^3^
370 parameters	Δρ_min_ = −0.35 e Å^−^^3^
0 restraints	Absolute structure: Flack (1983), 1839 Friedel pairs
Primary atom site location: structure-invariant direct methods	Flack parameter: 0.008 (8)

### Special details

**Table:**

Geometry. All e.s.d.'s (except the e.s.d. in the dihedral angle between two l.s. planes) are estimated using the full covariance matrix. The cell e.s.d.'s are taken into account individually in the estimation of e.s.d.'s in distances, angles and torsion angles; correlations between e.s.d.'s in cell parameters are only used when they are defined by crystal symmetry. An approximate (isotropic) treatment of cell e.s.d.'s is used for estimating e.s.d.'s involving l.s. planes.
Refinement. Refinement of *F*^2^ against ALL reflections. The weighted *R*-factor *wR* and goodness of fit *S* are based on *F*^2^, conventional *R*-factors *R* are based on *F*, with *F* set to zero for negative *F*^2^. The threshold expression of *F*^2^ > σ(*F*^2^) is used only for calculating *R*-factors(gt) *etc*. and is not relevant to the choice of reflections for refinement. *R*-factors based on *F*^2^ are statistically about twice as large as those based on *F*, and *R*- factors based on ALL data will be even larger.

### Fractional atomic coordinates and isotropic or equivalent isotropic displacement parameters (Å^2^)

**Table:**

	*x*	*y*	*z*	*U*_iso_*/*U*_eq_	
C36	0.8319 (4)	0.9667 (3)	0.15546 (12)	0.0480 (10)	
C37	0.7036 (5)	0.9418 (3)	0.12979 (13)	0.0504 (9)	
H37	0.6686	0.8689	0.1285	0.060*	
Br1	0.30618 (5)	0.68396 (4)	0.081628 (14)	0.06634 (15)	
Br2	0.94686 (6)	0.84669 (4)	0.180636 (15)	0.07790 (17)	
O3	0.8123 (3)	0.85002 (19)	0.03021 (8)	0.0485 (6)	
O2	0.8426 (3)	0.56317 (18)	0.05928 (8)	0.0462 (6)	
O1	0.6407 (3)	0.4291 (2)	0.11810 (8)	0.0476 (6)	
C20	0.7812 (4)	0.6565 (3)	0.03631 (12)	0.0462 (9)	
H20A	0.6803	0.6718	0.0480	0.055*	
H20B	0.7758	0.6439	0.0024	0.055*	
C32	0.7909 (5)	1.1572 (3)	0.14345 (13)	0.0608 (11)	
H32	0.8128	1.2307	0.1511	0.073*	
C1	0.3373 (4)	0.6550 (3)	0.14721 (12)	0.0445 (9)	
C22	0.8814 (4)	0.9506 (3)	0.03838 (12)	0.0409 (9)	
C19	0.7751 (4)	0.4601 (3)	0.04702 (14)	0.0482 (9)	
H19A	0.8415	0.4003	0.0571	0.058*	
H19B	0.7664	0.4560	0.0128	0.058*	
C21	0.8846 (4)	0.7518 (3)	0.04703 (13)	0.0446 (9)	
H21A	0.9023	0.7570	0.0809	0.054*	
H21B	0.9810	0.7415	0.0313	0.054*	
C5	0.2497 (5)	0.6113 (3)	0.22280 (14)	0.0529 (10)	
H5	0.1702	0.6053	0.2442	0.064*	
C6	0.2185 (4)	0.6201 (3)	0.17485 (13)	0.0431 (9)	
C13	0.2424 (4)	0.3949 (3)	0.15319 (14)	0.0470 (10)	
C27	0.7969 (5)	1.0433 (3)	0.02457 (12)	0.0493 (10)	
C33	0.8887 (4)	1.0734 (3)	0.15801 (12)	0.0520 (10)	
C14	0.3689 (4)	0.4055 (3)	0.12492 (13)	0.0437 (9)	
H14	0.3573	0.4127	0.0923	0.052*	
C15	0.5121 (4)	0.4056 (2)	0.14417 (12)	0.0377 (8)	
C3	0.5171 (4)	0.6198 (3)	0.20919 (14)	0.0495 (10)	
C2	0.4856 (4)	0.6531 (3)	0.16317 (13)	0.0474 (9)	
H2	0.5637	0.6741	0.1431	0.057*	
C31	0.6617 (5)	1.1336 (3)	0.11786 (15)	0.0604 (11)	
H31	0.5979	1.1910	0.1086	0.072*	
C24	1.0629 (4)	1.0686 (3)	0.07684 (14)	0.0545 (10)	
C30	0.6268 (4)	1.0250 (3)	0.10591 (14)	0.0523 (10)	
C10	0.5332 (4)	0.3950 (3)	0.19276 (13)	0.0463 (9)	
C12	0.2648 (5)	0.3616 (3)	0.19911 (15)	0.0576 (11)	
H12	0.1831	0.3380	0.2172	0.069*	
C18	0.6230 (4)	0.4419 (3)	0.06822 (12)	0.0457 (9)	
H18A	0.5580	0.5049	0.0615	0.055*	
H18B	0.5770	0.3757	0.0548	0.055*	
C26	0.8660 (5)	1.1451 (3)	0.02910 (14)	0.0617 (11)	
H26	0.8225	1.2067	0.0146	0.074*	
C25	0.9965 (5)	1.1587 (3)	0.05427 (15)	0.0631 (12)	
H25	1.0411	1.2286	0.0563	0.076*	
C23	1.0121 (4)	0.9619 (3)	0.06396 (13)	0.0447 (9)	
H23	1.0669	0.8993	0.0728	0.054*	
C4	0.3970 (5)	0.6113 (3)	0.23964 (14)	0.0573 (11)	
H4	0.4145	0.6054	0.2720	0.069*	
C34	1.0530 (5)	1.1017 (4)	0.16609 (16)	0.0777 (13)	
H34A	1.0907	1.0575	0.1923	0.093*	
H34B	1.0607	1.1794	0.1750	0.093*	
C9	0.6714 (5)	0.4413 (3)	0.21679 (14)	0.0637 (11)	
H9A	0.7605	0.4190	0.1992	0.076*	
H9B	0.6792	0.4098	0.2483	0.076*	
C28	0.6283 (5)	1.0321 (3)	0.01627 (15)	0.0649 (12)	
H28A	0.6112	0.9766	−0.0081	0.078*	
H28B	0.5893	1.1024	0.0047	0.078*	
C17	0.0757 (4)	0.5687 (3)	0.15545 (15)	0.0569 (10)	
H17A	0.0389	0.6144	0.1296	0.068*	
H17B	−0.0007	0.5688	0.1801	0.068*	
C35	1.1557 (4)	1.0805 (4)	0.12142 (17)	0.0720 (13)	
H35A	1.2257	1.1420	0.1178	0.086*	
H35B	1.2144	1.0132	0.1263	0.086*	
C8	0.6679 (5)	0.5712 (4)	0.22061 (18)	0.0763 (13)	
H8A	0.6958	0.5927	0.2525	0.092*	
H8B	0.7426	0.6021	0.1992	0.092*	
C11	0.4078 (5)	0.3627 (3)	0.21874 (14)	0.0540 (10)	
H11	0.4202	0.3413	0.2501	0.065*	
C29	0.5392 (5)	0.9983 (4)	0.06158 (16)	0.0708 (12)	
H29A	0.4434	1.0376	0.0622	0.085*	
H29B	0.5179	0.9192	0.0606	0.085*	
C16	0.0974 (4)	0.4463 (3)	0.13707 (16)	0.0604 (11)	
H16A	0.0138	0.4011	0.1481	0.073*	
H16B	0.0948	0.4465	0.1027	0.073*	

### Atomic displacement parameters (Å^2^)

**Table:**

	*U*^11^	*U*^22^	*U*^33^	*U*^12^	*U*^13^	*U*^23^
C36	0.067 (3)	0.041 (2)	0.035 (2)	0.0044 (19)	0.0096 (19)	−0.0025 (16)
C37	0.061 (3)	0.045 (2)	0.045 (2)	−0.008 (2)	0.015 (2)	−0.0008 (18)
Br1	0.0764 (3)	0.0724 (3)	0.0503 (2)	0.0140 (2)	0.0005 (2)	0.0209 (2)
Br2	0.1185 (4)	0.0657 (3)	0.0495 (2)	0.0250 (3)	−0.0064 (2)	0.0065 (2)
O3	0.0626 (15)	0.0360 (14)	0.0469 (14)	0.0062 (13)	−0.0120 (12)	−0.0028 (11)
O2	0.0512 (15)	0.0357 (13)	0.0518 (15)	0.0001 (11)	−0.0107 (11)	0.0038 (11)
O1	0.0444 (15)	0.0576 (16)	0.0408 (15)	0.0075 (12)	−0.0015 (11)	0.0014 (12)
C20	0.050 (2)	0.041 (2)	0.047 (2)	0.0013 (18)	−0.0043 (17)	0.0023 (17)
C32	0.093 (3)	0.041 (2)	0.048 (2)	0.003 (3)	0.020 (2)	−0.0084 (19)
C1	0.057 (3)	0.0297 (19)	0.046 (2)	0.0067 (17)	0.0024 (18)	−0.0007 (16)
C22	0.062 (2)	0.032 (2)	0.0288 (19)	−0.0007 (17)	0.0063 (17)	−0.0032 (15)
C19	0.051 (2)	0.043 (2)	0.051 (2)	0.0000 (17)	0.0003 (18)	−0.0058 (17)
C21	0.055 (2)	0.036 (2)	0.043 (2)	0.0076 (17)	−0.0055 (18)	−0.0001 (16)
C5	0.064 (3)	0.053 (2)	0.042 (2)	0.0063 (18)	0.0116 (19)	−0.0043 (18)
C6	0.041 (2)	0.0379 (19)	0.051 (2)	0.0099 (16)	0.0042 (19)	−0.0009 (16)
C13	0.044 (2)	0.037 (2)	0.059 (3)	−0.0099 (16)	0.0070 (19)	−0.0029 (18)
C27	0.082 (3)	0.032 (2)	0.034 (2)	0.007 (2)	0.005 (2)	0.0003 (15)
C33	0.064 (3)	0.055 (3)	0.036 (2)	−0.002 (2)	0.0006 (18)	−0.0110 (17)
C14	0.053 (2)	0.030 (2)	0.048 (2)	−0.0042 (16)	−0.0032 (19)	−0.0041 (16)
C15	0.043 (2)	0.0244 (18)	0.046 (2)	0.0044 (15)	0.0017 (17)	0.0036 (15)
C3	0.048 (3)	0.043 (2)	0.057 (3)	−0.0039 (17)	−0.011 (2)	−0.0122 (17)
C2	0.046 (2)	0.040 (2)	0.056 (2)	−0.0076 (17)	0.0028 (18)	−0.0023 (18)
C31	0.073 (3)	0.054 (3)	0.054 (3)	0.023 (2)	0.012 (2)	−0.003 (2)
C24	0.052 (2)	0.051 (2)	0.060 (3)	−0.0082 (19)	0.018 (2)	−0.008 (2)
C30	0.050 (2)	0.051 (3)	0.056 (3)	0.0055 (19)	0.0150 (19)	−0.005 (2)
C10	0.054 (2)	0.0371 (19)	0.048 (2)	0.0125 (17)	−0.004 (2)	0.0043 (16)
C12	0.064 (3)	0.044 (2)	0.065 (3)	−0.0105 (19)	0.019 (2)	0.003 (2)
C18	0.046 (2)	0.047 (2)	0.044 (2)	−0.0029 (17)	−0.0041 (17)	−0.0017 (17)
C26	0.101 (4)	0.043 (3)	0.041 (2)	0.002 (2)	0.011 (2)	0.0051 (19)
C25	0.090 (3)	0.034 (2)	0.066 (3)	−0.019 (2)	0.031 (2)	−0.002 (2)
C23	0.048 (2)	0.040 (2)	0.046 (2)	0.0025 (16)	0.0140 (18)	−0.0039 (16)
C4	0.075 (3)	0.056 (3)	0.041 (2)	0.005 (2)	−0.008 (2)	−0.0141 (18)
C34	0.087 (3)	0.076 (3)	0.070 (3)	−0.012 (3)	−0.012 (3)	−0.023 (2)
C9	0.067 (3)	0.078 (3)	0.046 (2)	0.023 (2)	−0.015 (2)	0.004 (2)
C28	0.081 (3)	0.052 (3)	0.062 (3)	0.019 (2)	−0.023 (2)	0.001 (2)
C17	0.039 (2)	0.066 (3)	0.066 (3)	0.0039 (19)	0.0009 (19)	0.000 (2)
C35	0.052 (3)	0.072 (3)	0.091 (4)	−0.011 (2)	−0.001 (2)	−0.023 (3)
C8	0.055 (3)	0.092 (4)	0.082 (3)	−0.009 (3)	−0.028 (2)	−0.007 (3)
C11	0.080 (3)	0.036 (2)	0.046 (2)	0.007 (2)	0.003 (2)	0.0136 (17)
C29	0.057 (3)	0.075 (3)	0.080 (3)	0.015 (2)	−0.011 (2)	−0.010 (2)
C16	0.043 (2)	0.063 (3)	0.075 (3)	−0.015 (2)	0.000 (2)	−0.005 (2)

### Geometric parameters (Å, °)

**Table:**

C36—C33	1.380 (5)	C3—C2	1.388 (5)
C36—C37	1.380 (5)	C3—C8	1.492 (5)
C36—Br2	1.903 (4)	C2—H2	0.9300
C37—C30	1.384 (5)	C31—C30	1.383 (5)
C37—H37	0.9300	C31—H31	0.9300
Br1—C1	1.905 (3)	C24—C25	1.387 (6)
O3—C22	1.374 (4)	C24—C23	1.407 (5)
O3—C21	1.424 (4)	C24—C35	1.510 (6)
O2—C20	1.405 (4)	C30—C29	1.507 (6)
O2—C19	1.418 (4)	C10—C11	1.386 (5)
O1—C15	1.385 (4)	C10—C9	1.506 (5)
O1—C18	1.426 (4)	C12—C11	1.381 (6)
C20—C21	1.497 (5)	C12—H12	0.9300
C20—H20A	0.9700	C18—H18A	0.9700
C20—H20B	0.9700	C18—H18B	0.9700
C32—C31	1.382 (6)	C26—C25	1.366 (6)
C32—C33	1.390 (6)	C26—H26	0.9300
C32—H32	0.9300	C25—H25	0.9300
C1—C6	1.375 (5)	C23—H23	0.9300
C1—C2	1.387 (5)	C4—H4	0.9300
C22—C23	1.370 (5)	C34—C35	1.576 (6)
C22—C27	1.397 (5)	C34—H34A	0.9700
C19—C18	1.490 (5)	C34—H34B	0.9700
C19—H19A	0.9700	C9—C8	1.565 (6)
C19—H19B	0.9700	C9—H9A	0.9700
C21—H21A	0.9700	C9—H9B	0.9700
C21—H21B	0.9700	C28—C29	1.557 (6)
C5—C6	1.386 (5)	C28—H28A	0.9700
C5—C4	1.387 (5)	C28—H28B	0.9700
C5—H5	0.9300	C17—C16	1.572 (5)
C6—C17	1.509 (5)	C17—H17A	0.9700
C13—C12	1.372 (5)	C17—H17B	0.9700
C13—C14	1.381 (5)	C35—H35A	0.9700
C13—C16	1.495 (5)	C35—H35B	0.9700
C27—C26	1.373 (5)	C8—H8A	0.9700
C27—C28	1.517 (6)	C8—H8B	0.9700
C33—C34	1.510 (6)	C11—H11	0.9300
C14—C15	1.379 (5)	C29—H29A	0.9700
C14—H14	0.9300	C29—H29B	0.9700
C15—C10	1.391 (5)	C16—H16A	0.9700
C3—C4	1.372 (5)	C16—H16B	0.9700
			
C33—C36—C37	121.9 (3)	C15—C10—C9	121.3 (3)
C33—C36—Br2	119.4 (3)	C13—C12—C11	120.6 (3)
C37—C36—Br2	118.2 (3)	C13—C12—H12	119.7
C36—C37—C30	120.3 (3)	C11—C12—H12	119.7
C36—C37—H37	119.9	O1—C18—C19	108.3 (3)
C30—C37—H37	119.9	O1—C18—H18A	110.0
C22—O3—C21	118.3 (3)	C19—C18—H18A	110.0
C20—O2—C19	115.0 (3)	O1—C18—H18B	110.0
C15—O1—C18	117.2 (3)	C19—C18—H18B	110.0
O2—C20—C21	106.4 (3)	H18A—C18—H18B	108.4
O2—C20—H20A	110.5	C25—C26—C27	122.1 (4)
C21—C20—H20A	110.5	C25—C26—H26	118.9
O2—C20—H20B	110.5	C27—C26—H26	118.9
C21—C20—H20B	110.5	C26—C25—C24	120.3 (4)
H20A—C20—H20B	108.6	C26—C25—H25	119.9
C31—C32—C33	121.4 (4)	C24—C25—H25	119.9
C31—C32—H32	119.3	C22—C23—C24	119.8 (4)
C33—C32—H32	119.3	C22—C23—H23	120.1
C6—C1—C2	122.2 (3)	C24—C23—H23	120.1
C6—C1—Br1	119.8 (3)	C3—C4—C5	120.9 (4)
C2—C1—Br1	117.1 (3)	C3—C4—H4	119.6
C23—C22—O3	123.4 (3)	C5—C4—H4	119.6
C23—C22—C27	121.3 (3)	C33—C34—C35	113.5 (3)
O3—C22—C27	114.6 (3)	C33—C34—H34A	108.9
O2—C19—C18	114.2 (3)	C35—C34—H34A	108.9
O2—C19—H19A	108.7	C33—C34—H34B	108.9
C18—C19—H19A	108.7	C35—C34—H34B	108.9
O2—C19—H19B	108.7	H34A—C34—H34B	107.7
C18—C19—H19B	108.7	C10—C9—C8	112.5 (3)
H19A—C19—H19B	107.6	C10—C9—H9A	109.1
O3—C21—C20	107.0 (3)	C8—C9—H9A	109.1
O3—C21—H21A	110.3	C10—C9—H9B	109.1
C20—C21—H21A	110.3	C8—C9—H9B	109.1
O3—C21—H21B	110.3	H9A—C9—H9B	107.8
C20—C21—H21B	110.3	C27—C28—C29	113.2 (3)
H21A—C21—H21B	108.6	C27—C28—H28A	108.9
C6—C5—C4	121.5 (4)	C29—C28—H28A	108.9
C6—C5—H5	119.3	C27—C28—H28B	108.9
C4—C5—H5	119.3	C29—C28—H28B	108.9
C1—C6—C5	115.2 (3)	H28A—C28—H28B	107.7
C1—C6—C17	124.0 (3)	C6—C17—C16	113.6 (3)
C5—C6—C17	119.4 (3)	C6—C17—H17A	108.8
C12—C13—C14	117.2 (4)	C16—C17—H17A	108.8
C12—C13—C16	122.2 (4)	C6—C17—H17B	108.8
C14—C13—C16	118.8 (3)	C16—C17—H17B	108.8
C26—C27—C22	116.5 (4)	H17A—C17—H17B	107.7
C26—C27—C28	122.1 (4)	C24—C35—C34	111.6 (3)
C22—C27—C28	119.9 (3)	C24—C35—H35A	109.3
C36—C33—C32	115.5 (4)	C34—C35—H35A	109.3
C36—C33—C34	124.6 (4)	C24—C35—H35B	109.3
C32—C33—C34	118.8 (4)	C34—C35—H35B	109.3
C15—C14—C13	121.1 (3)	H35A—C35—H35B	108.0
C15—C14—H14	119.4	C3—C8—C9	113.2 (3)
C13—C14—H14	119.4	C3—C8—H8A	108.9
C14—C15—O1	123.1 (3)	C9—C8—H8A	108.9
C14—C15—C10	120.8 (3)	C3—C8—H8B	108.9
O1—C15—C10	115.6 (3)	C9—C8—H8B	108.9
C4—C3—C2	116.9 (3)	H8A—C8—H8B	107.7
C4—C3—C8	121.9 (4)	C12—C11—C10	121.6 (4)
C2—C3—C8	119.7 (4)	C12—C11—H11	119.2
C1—C2—C3	120.0 (3)	C10—C11—H11	119.2
C1—C2—H2	120.0	C30—C29—C28	111.5 (3)
C3—C2—H2	120.0	C30—C29—H29A	109.3
C32—C31—C30	120.4 (4)	C28—C29—H29A	109.3
C32—C31—H31	119.8	C30—C29—H29B	109.3
C30—C31—H31	119.8	C28—C29—H29B	109.3
C25—C24—C23	117.2 (4)	H29A—C29—H29B	108.0
C25—C24—C35	122.7 (4)	C13—C16—C17	113.0 (3)
C23—C24—C35	118.4 (4)	C13—C16—H16A	109.0
C31—C30—C37	116.9 (4)	C17—C16—H16A	109.0
C31—C30—C29	121.2 (4)	C13—C16—H16B	109.0
C37—C30—C29	120.3 (4)	C17—C16—H16B	109.0
C11—C10—C15	116.1 (3)	H16A—C16—H16B	107.8
C11—C10—C9	121.0 (3)		

### Hydrogen-bond geometry (Å, °)

**Table:**

*D*—H···*A*	*D*—H	H···*A*	*D*···*A*	*D*—H···*A*
C17—H17A···O2^i^	0.97	2.71	3.412 (5)	130

Symmetry codes: (i) *x*−1, *y*, *z*.
